# Prospect of radiotherapy technology development in the era of immunotherapy

**DOI:** 10.1016/j.jncc.2022.04.001

**Published:** 2022-04-29

**Authors:** Jian-Yue Jin

**Affiliations:** Radiation Oncology, Seidman Cancer Center, University Hospitals, Case Western Reserve University, Cleveland, United States

**Keywords:** Radiotherapy technology, Immune sparing technique, Proton therapy, FLASH radiotherapy, Spatially fractionated radiotherapy

## Abstract

Radiotherapy (RT) is one of the important modalities for cancer treatments. Mounting evidence suggests that the host immune system is involved in the tumor cell killing during RT, and future RT technology development should aim to minimize radiation dose to the immune system while maintaining a sufficient dose to the tumor. A brief history of RT technology development is first summarized. Three RT technologies, namely FLASH RT, proton therapy, and spatially fractionated RT (SFRT), are singled out for the era of immunotherapy. Besides the technical aspects, the mechanism of FLASH effect is discussed, which is likely the combined results of the recombination effect, oxygen depletion effect and immune sparing effect. The proton therapy should have the advantage of causing much less immune damage in comparison to X-ray based RT due to the Bragg peak. However, the relative biological effectiveness (RBE) uncertainty and range uncertainty may hinder the translation of this advantage into clinical benefit. Research approaches to overcome these two technical hurdles are discussed. Various SFRT approaches and their application are reviewed. These approaches are categorized as single-field 1D/2D SFRT, multi-field 3D SFRT and quasi-3D SFRT techniques. A 3D SFRT approach, which is achieved by placing the Bragg peak of a proton 2D SFRT field in discrete depths, may have special potential because all 3 technologies (FLASH RT, proton therapy and SFRT) may be used in this approach.

## 1 Introduction

Radiotherapy (RT) is one of the important modalities for cancer treatments. Approximately two thirds of the cancer patients would require radiotherapy as part of their cancer treatments.^1^ Since the first use of RT more than 125 years ago,^2^ the RT technology has undergone tremendous progress. These technological improvements were mainly aimed to maximize radiation dose to the target (tumor), while minimizing radiation dose to the surrounding critical organs. According to the basic radiobiology theory, increasing radiation dose to the tumor increases the chance of tumor cell killing and hence the tumor control, while increasing the dose to the normal organs increases the chance of normal tissue toxicities. Therefore, technological improvements have the potential to increase the therapeutic ratio or treatment outcome by increasing tumor control and/or reducing normal tissue toxicities. Long term clinical data have supported that the technological development in RT have been translated into improvement of clinical outcomes.^3^

Recently, studies on cancer immunology, especially the success of clinical trials in cancer immunotherapy, have established the important role of immune system in cancer treatments.^4^, ^5^ Studies also support a direct role of immune system in cancer radiotherapy.^6^, ^7^, ^8^, ^9^, ^10^, ^11^, ^12^, ^13^, ^14^, ^15^, ^16^ Reports of the abscopal effect,^6^ partial tumor irradiation^7^, ^8^ and spatially fractionated radiotherapy (SFRT)^9^ indicate a radiation-induced immune effect on un-irradiated tumor cells in distant metastatic tumors or within the irradiated primary tumor itself. In addition, tumor *in vivo* radiosensitivity appeared to depend on the genetic difference between the tumor and the host.^10^, ^11^, ^12^ Tumors were much more radiosensitive when they were implanted in hybrid mice than in mice of an original strain from which the tumors came,^10^, ^11^ suggesting that the immune system in hybrid mice recognizes the tumor cells as “foreign invaders” more than the immune system in mice of the original strain. Mutated tumors were more radiosensitive than un-mutated ones when they were all implanted in mice of an original strain.^12^ Furthermore, tumors were more radiosensitive in mice with an intact immune system than in mice with a compromised immune system, such as in the nude mice^13^ or mice with the immune system damaged by total body irradiation (TBI).^14^, ^15^ Recent studies have also reported potential mechanisms of radiation-activated antitumor immunity.^16^, ^17^, ^18^, ^19^ For example, it was found that radiation killing of tumor cells generates abundance of double strand DNAs (dsDNA) in the cytosol,^16^ and the cyclic GMP-AMP (cGAMP) synthase (cGAS) senses the dsDNAs in the cytosol,^17^, ^18^ which activates the stimulation of interferon genes (STING),^17^, ^18^ which further stimulates the secretion of type I interferon and finally activates the adoptive immune response.^16^, ^17^, ^18^ It was also reported that radiation induced release of programmed cell death-ligand 1 (PD-L1) molecules from the tumor.^19^

However, RT also delivers incidental radiation doses to the surrounding normal tissues, including lymphatic tissues and bone marrow, as well as to the circulating immune cells in blood that passes through the radiation field, which may partially damage the immune system. It has been well established that severe damage of the immune system by TBI greatly degraded tumor radiosensitivity.^14^, ^15^ Studies also suggest that partial damage of the host immune system by incidental radiation during RT may degrade the tumor radiosensitivity.^20^, ^21^, ^22^, ^23^, ^24^, ^25^, ^26^, ^27^ It was reported that much higher radiation dose was required to eliminate tumor cell *in vivo*, in comparison to a mixed situation with irradiation of tumor cells *in vitro* and implanting the irradiated tumor cells into mice.^20^ In the *in vivo* case, the irradiation of tumor might partially damage the immune system, while in the mixed situation, the immune system was intact because the irradiation was *in vitro*. Partial irradiation in the abdominal region (which might have partially damaged the immune system) significantly reduced the anti-tumor immunity as demonstrated by increasing the take of tumor cell implant in the thoracic region in comparison to no radiation.^21^ While a single dose of 30 Gy controlled a tumor in a mouse, adding additional 10 doses of 3 Gy to the tumor could not control the tumor.^22^ In an animal study of high-dose-rate brachytherapy comparing doses of 2, 5, 10 and 15 Gy, 10 and 15 Gy appeared to well control a tumor, with 10 Gy slightly better than 15 Gy, and 10 Gy enhanced CD8^+^ cytotoxic lymphocyte infiltration in the irradiated tumor by 7 folds, while 5 Gy and 15 Gy only enhanced the infiltration by 2 folds.^23^ These data suggest that overdose may suppress activated immune cells and degrade tumor control. Clinical studies also showed radiation induced lymphopenia after RT, and patients with severe lymphopenia had poorer survival.^24^ An estimated effective dose to immune cells in circulating blood (EDIC) was reported to correlate with survival^25^, ^26^, ^27^ as well as to lymphopenia.^26^, ^27^

These data are in contrast with the conventional radiobiology that believes increasing the dose to a tumor would always increase the tumor control. An overdose to the tumor may unnecessarily increase the dose to the immune system, damage the immune system, and hence reduce the tumor control. This disruptive change of the fundamental radiobiology may greatly impact the future development of the RT technologies. Specifically, future RT technologies would aim to minimize the radiation dose to the immune system while maintaining a sufficient dose to the tumor. In this paper, we will first review the history of the technological progress before the immunotherapy era, and then discuss three RT technologies that can achieve the goal of minimizing the dose to the immune system. These technologies include 1) FLASH RT, 2) proton therapy, and 3) SFRT.

## 2 RT technology progression before the immunotherapy era

The progressions of RT technologies have been mainly aimed to minimize the radiation dose to the surrounding critical organs while maximizing the dose to the tumor, and they are reflected in the following four fronts: 1) improvement of radiation sources, 2) improvement of radiation delivery, 3) development of a treatment planning system, and 4) development of imaging guidance technology in the treatment machine.

### 2.1 Improvement of radiation sources

The improvement of radiation sources was from low-energy photon radiation sources with shallow penetration to high-energy photon sources with deep penetration, and to proton and heavy-ions. Low-energy X-rays were first used in breast cancer treatment on January 29, 1896.^2^^,^^28^ Low-energy X-ray machines and natural radioactive isotopes such as Radium-226/228 were frequently used for treating various cancers until 1930s. However, as these radiation sources had very shallow penetration, they could only treat skin cancers effectively. The manufacture of relatively higher energy orthovoltage (200-500 keV) X-ray devices started an orthovoltage era in 1930s-1950s. On March 1, 1937, the first Van de Graaff accelerator based MV X-ray machine was used to treat patients.^28^ On October 27, 1951, a Co-60 radioactive-isotope-based radiotherapy device, which emitted relatively high-energy gamma rays (1.17 and 1.33 MeV), was first used clinically, and gradually replaced the orthovoltage X-rays and Van de Graaff machines.^28^ The first linear accelerator (LINAC)-based X-ray RT machine started treating patients on September 7, 1953.^29^ The LINACs with X-ray energies of 6-18 MV, and electron beam energies of 6-22 MeV have gradually replaced Co-60 machines and become the mainstream RT modality. Meantime, expensive proton and heavy-ion RT machines have been developed to treat patients due to their dosimetric advantage over the X-rays.

### 2.2 Improvement of radiation delivery

The improvement of radiation delivery is mainly reflected by 1) use of accelerators to generate the radiation, instead of radiation from isotopes, so that the radiation can be easily shut down by turning off the accelerators; 2) use of multiple radiation fields, especially multi-field stereotactic non-coplanar radiation delivery, which can greatly improve the dose conformity to the target; 3) use of multi-leave collimators (MLC) for quick collimator shaping to conform with the target, and for intensity modulated radiotherapy (IMRT) to spare critical organs while maintaining required dose to the target; and 4) arc delivery and intensity modulated arc therapy.

### 2.3 Development of a treatment planning system

A treatment planning system was initially developed to do simple dose calculations in 1970s. The system was significantly improved with the use of computed tomography (CT) images. Currently, the most sophisticated treatment planning system can include the following major capabilities: 1) model the patient body using CT or other imaging modalities; 2) delineate the tumor as well as critical organs in the imaging modality; 3) model the radiation treatment machine for accurate calculation of the 3-dimentional dose distribution in the tumor and organs; 4) set and adjust the radiation fields with their dose, energy, direction, and position for optimization of the treatment plan; 5) use inverse planning algorithms for IMRT to achieve desired dose distribution to the tumor and critical organs, and 6) use machine learning or other artificial intelligence (AI) algorithms to optimize various treatment parameters.

### 2.4 Development of image-guidance technology in a treatment machine

Electronic portal image device (EPID) was first integrated into a treatment machine in 1990s to replace portal films as verification of treatment position.^30^ Based on 2D-3D imaging registration, a pair of orthogonal 2D X-ray imagers was first used as 2D imaging guidance for accurate patient positioning.^31^ Currently, most linear accelerators are equipped with a 3D cone beam CT imager, which provides 3D image guidance with 3D-3D image registration.^32^ Treatment machines integrated with a MRI have also gained wide acceptance due to their ability for soft tissue visualization during treatment.^33^ A positron emission tomography (PET)/CT integrated treatment machine has also just been developed.^34^ The use of these integrated 3D imaging modalities in the treatment machine not only greatly improved the position accuracy, but also opened the door for image-guided adaptive therapy.

## 3 FLASH radiotherapy

FLASH RT is a technique that has great potential in sparing immune system during RT. It delivers ultra-fast radiation treatment with a dose rate several orders of magnitude higher than conventional dose rates.^35^, ^36^ It has been reported that FLASH RT can significantly spare normal tissues in comparison to RT at conventional dose rates, whereas tumor responses are the same as or better than those resulting from conventional dose rate RT.^35^, ^36^ Because the mechanism of the FLASH effect is still not well established, here, we focus on the discussion of potential mechanisms as well as the technological development to achieve FLASH dose rate.

### 3.1 Mechanism of the FLASH effect

Studies suggest that the experimentally observed FLASH effect is likely to be the combined results of three effects^37^, ^38^, ^39^, ^40^, ^41^: 1) the recombination effect, 2) the oxygen depletion effect, and 3) the immune sparing effect.

The recombination effect refers to the increase of the recombination of radiation-induced free radicals when the radiation dose rate is extremely high. Two free radicals may encounter each other and recombine into a harmless molecule before they impose a damage to a DNA. Increase of dose rate increases the chance of recombination, decreases the number of free radicals and hence reduce the radiation effect. *Labarbe et al.* did a simulation study of the recombination effect, and found that when the dose rate increased to 30-50 Gy/second, the damage to the normal tissue reduced to 50% of that in conventional dose rate.^37^ *Jansen et al.* measured oxygen consumption per 10 Gy radiation in a closed water phantom as a surrogate for free radical recombination, and found that the number of recombination increased, or the remaining number of free radicals reduced with increasing dose rate,^38^ and experimentally confirmed the recombination effect. As *Jansen et al.* demonstrated, the recombination effect occurs in water. Therefore, it may occur in both normal cells and cancer cells.

The oxygen depletion effect is based on the assumption that oxygen is consumed by radiation in forming free radicals, and at ultra-high dose rate, the oxygen consumption rate is much higher than the oxygen supply rate so that a transient hypoxic status is generated. The hypoxic status reduces the radiation killing effect. Pratx and Kapp did a simulation study and found that the oxygen consumption by 10 Gy of radiation would not induce meaningful change of radiation effect if the oxygen tension was larger than 15 mmHg.^39^ Only when the cells were in an extreme hypoxic condition (for example, oxygen tension < 5-8 mmHg), the oxygen depletion may induce a significant sparing effect. *Jansen et al.* also did experimental measurements, and found that it took about more than 150 Gy to reduce the oxygen concentration from 10% air pressure to 9% air pressure, indicating oxygen depletion has minimal effect on the sparing of radiation damages.^38^

**Fig. 1:**
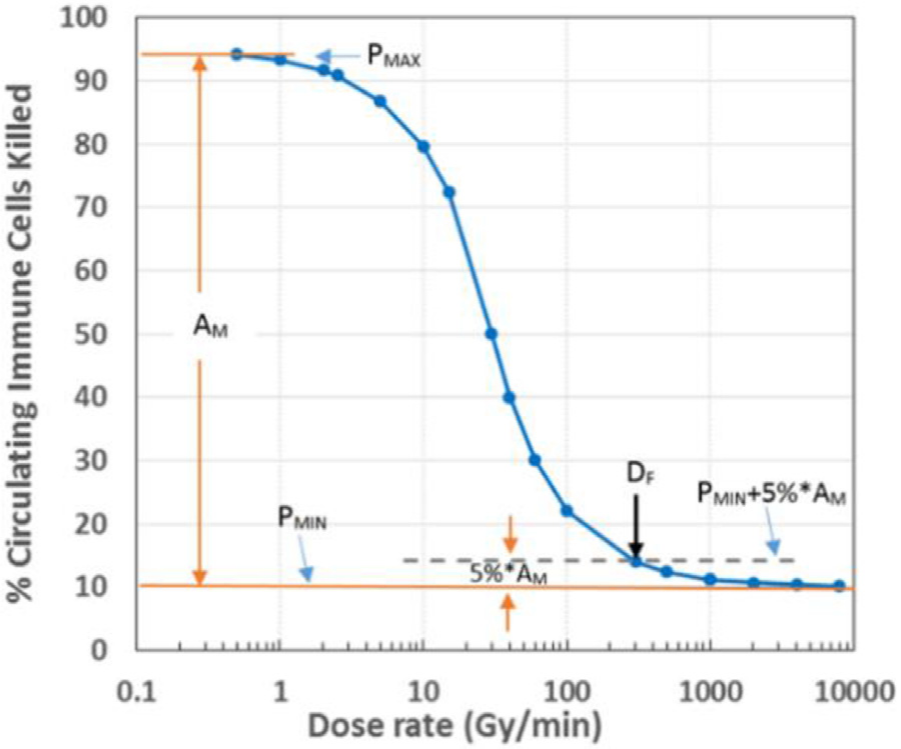
Sparing of circulating immune cells in the blood by FLASH RT according to a simulation study by Jin et al.^40^ Assuming that the irradiated volume contains 10% total blood, irradiation dose is 30 Gy, the blood have a circulation cycle of 1 minute. The percentage of circulating immune cells is ∼94% when the dose rate is < 2 Gy/minute, and drops to ∼10% when the dose rate increases to 2000 Gy/minute.

The immune sparing effect refers to the sparing of circulating immune cells in the blood by FLASH radiation. Under the FLASH RT condition, the irradiation time is so short that only a small portion of blood flows into the radiation field, whereas in conventional RT, the irradiation time can last several minutes (especially for large dose per fraction in hypofractionated treatments) so that the blood may flow into the radiation field for several cycles. *Jin et al.* did a simulation study in various scenarios and found that the percentage killed immune cells in circulation blood versus the dose rate showed a sigmoid-shaped curve, as illustrated in an example with 30 Gy radiation to a volume containing 10% of blood^40^ (Fig. 1). The radiation appears to kill ∼94% of the immune cells in the blood in conventional dose rate (<2 Gy/minute), while the same dose may only kill ∼10% of the immune cells under FLASH RT, assuming the blood circulation cycle is 1 minute. However, it should be noted that FLASH RT can only spare immune cells in circulating blood. The majority of immune cells may be stationed in other immune structures such as lymph nodes/ducts, spleen, bone marrow and other lymphatic tissues.^41^

In summary, these studies indicate that FLASH RT indeed can spare radiation damage to any cells due to the recombination effect, spare cells that are extremely hypoxic due to the oxygen depletion effect, and spare the immune cells in circulating blood due to the immune sparing effect. The sparing of immune cells in circulating blood may have profound effects on both tumor control and normal tissue toxicity. The FLASH effect has been mostly reported as sparing of normal tissues in various organs and species. The recombination effect may mainly contribute to these observed phenomena. Studies also reported that FLASH RT not only spared normal tissues, but also had the same or better effect on tumor control. The immune sparing effect may contribute to the reported improvement of tumor control.

### 3.2 Development of FLASH RT technology

The recombination effect may have minimal benefit to RT because it can spare both tumor and normal cells. However, the immune sparing effect may be utilized to improve the RT outcome. Considering the blood circulation cycle is about 1 minute in human beings, the radiation delivery time of 5-10 seconds would spare significant immune cells in blood in comparison to conventional dose-rate RT, which typically requires several minutes. Therefore, the required dose rate is about 2-4 Gy/second (120-240 Gy/minute) if the maximal dose per fraction is 20 Gy. The dose rate of X-rays from a 15 MV linear accelerator may reach 50-100 Gy/minute at the 100 cm source to target distance. Therefore, an X-ray based FLASH RT machine can be developed with a combination of three linear accelerators. A conceptual FLASH RT machine named PHASER, which is composed of 12 linear accelerators, has been proposed.^42^

A single-source FLASH RT machine can also be developed. A clinical proton machine can be easily converted into a FLASH RT proton unit with a dose rate > 100 Gy/second.^43^ The electron beams from the clinical linear accelerators can also be converted into FLASH RT machines. However, the penetration of these electrons are not sufficient to treat deep-seated tumors, so they have been mostly used for pre-clinical studies. A very high-energy electron beam (100-250 MeV) has also been proposed as a single source FLASH unit.^44^ In addition, it was reported that high-energy X-rays (6-7 MeV) from a superconductor-based LINAC could generate >100 Gy/second FLASH radiation for preclinical studies.^45^ However, this experiment was possibly performed with a source target distance of 8-15 cm. The dose rate at clinically useful source target distance (∼100 cm) might be in the order of 1 Gy/second.

It should also be noted that, clinically, multiple radiation fields are often used to treat a tumor so that the radiation can be conformed to the tumor. However, for a single radiation source, it often takes relatively longer time for the source to move from one field to another. New blood may flow into the tumor region when the next field is irradiated. The number of irradiated immune cells may be multiplied with multiple fields unless an arc delivery is used. Therefore, a rapid arc-delivery within 5-10 seconds should be considered for single-source FLASH RT. In the situation of multiple-source irradiation, the tumor is simultaneously irradiated by the crossfire of several sources, while the surrounding normal tissues may be only irradiated by one source. The dose rate to the tumor may be several times higher than that to the normal tissues. Consequently, the recombination effect may spare much more tumor cells than normal cells. Therefore, when multiple-source design is used, the dose rate should not be too high to induce significant recombination effect in tumor cells.

## 4 Proton/heavy ion radiotherapy

Proton and heavy-ion RT have unique dosimetric advantage over the conventional photon or electron-based RT because of the presence of the Bragg peak. The heavy-ion RT can be considered as a large scale proton machine. For simplicity, here we focus on the proton RT and the content regarding the proton RT may apply to heavy-ion RT. In theory, the Bragg peak means no exit dose (the dose in the outside target region after the radiation beam exits the target), and much lower entrance dose (the dose in the outside target region before the beam enters the target) in comparison to photon-based radiation. Therefore, the integral dose for proton RT should be much less than that for photon-based RT. Consequently, proton RT should induce much less immune damage than corresponding photon RT and thus be an ideal technological option in the era of immunotherapy. Indeed, it was reported that proton RT showed better clinical outcome than corresponding X-ray based RT in treating esophageal cancer.^46^ However, this clinic advantage has not been demonstrated in many other disease sites such as lung cancer. Two major technological hurdles, the relative biological effectiveness (RBE) uncertainty and the range uncertainty, may be the major causes that hinder the translation of the dosimetric advantage into clinical benefit. Here we focus on the research and technology development efforts to overcome these two hurdles.

### 4.1 RBE in proton therapy

Protons deposit much higher density of energy (or dose) along their tracks than photons, especially in the region of Bragg peak. These high-density dose depositions along the tracks generate clustered DNA damages that are much more difficult to be repaired. Therefore, the same average dose for protons induces relatively higher biological damage than that for the photons. The RBE has been defined to quantify this difference. However, due to the difficulty to model the complicated dependence of RBE on proton linear energy transfer (LET) (or location), dose, and cell type, a constant RBE value of 1.10 has been used in current clinical practice. This constant RBE has made the dose calculation in proton treatment plan highly unreliable and planning optimization difficult. Tremendous efforts have been devoted to develop a reliable RBE model. A large amount of experimental data have been accumulated in a Particle Irradiation Data Ensemble (PIDE) database.^47^ Empirical models that estimate the RBE from LET based on the published experimental data have been developed.^48^ Mechanistic models that originally developed for carbon therapy have also been applied for proton.^49^ These models appear to work well for the RBE dependence on LET and are superior to the constant RBE model of RBE=1.10. However, these models were less well tested for the dependence of cell type.

*Jin et al.* proposed that the unclear relationships of the two parameters (α and β) in the classic linear-quadratic (LQ) model with the LET have made it difficult to uncover the RBE model with LET, dose, and cell type from the experimental data.^50^ A novel cell survival model with two parameters representing the ability to generate damage by radiation (α) and the ability to repair the damage by cells (β) was proposed.^50^ According to the Poisson equation, the survival fraction (SF) of cells after a radiation dose of D can be expressed as:

$$SF\left(D\right)=\left(1+\alpha \beta D+\frac{\alpha^{2}\beta^{2}D^{2}}{2}+\frac{\alpha^{3}\beta^{3}D^{3}}{6}+\frac{\alpha^{4}\beta^{4}D^{4}}{24}+\frac{\alpha^{5}\beta^{5}D^{5}}{120}\right)*Exp\left(-\alpha D\right)$$

**Fig. 2:**
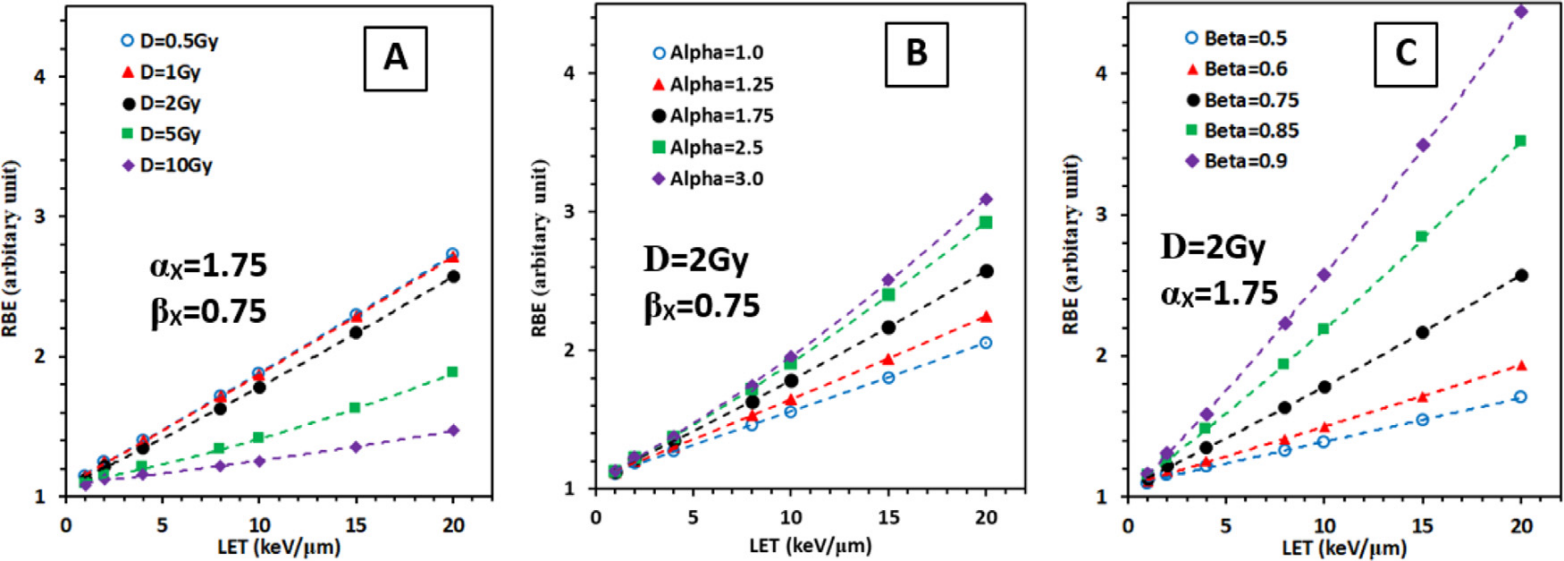
A relative RBE model developed by Jin et al.^50^ from experimental data collected in a database. (A) RBE varies with LET and dose/fraction (D) with a fixed cell type represented by two parameters (α_X_, β_X_), which correspond to the ability to generate damage by radiation, and the ability to repair the damage by the cell. (B) RBE varies with LET and α_X_ with fixed D and β_X_. (C) RBE varies with LET and β_X_ with fixed D and α_X_. LET, linear energy transfer; RBE, relative biological effectiveness.

The two parameters (α and β) were assumed to have a linear relationship with the LET according to their definition. Indeed, a unified survival model that can fit all the eligible experimental data in the PIDE database with various LETs, doses, and cell types were determined, and consequently a unified RBE model depending on LET, dose, and cell type was derived. As shown in Fig. 2, the RBE increases with LET for various doses/fraction and cell types. Assuming α_x_ and β_X_ are two parameters in our survival model that define the characteristics of cell type in X-ray irradiation, the RBE increases with LET more rapidly when the dose/fraction is low (0.5-1 Gy/fraction) (Fig. 2A), the α_X_ value is large (Fig. 2B), and the β_X_ value is large (Fig. 2C). The RBE reaches to 4.2 when LET = 20 keV/μm, α_X_ = 1.75 Gy^−1^, β_X_ = 0.9 Gy^−2^ and at 2 Gy/fraction. This is substantially larger than the 1.10 value used in clinics, suggesting that the dose calculation in current proton therapy planning system is highly unreliable, especially at the distal edge of the Bragg peak, for low dose/fraction, and for radiosensitive cell types. Future work would be needed to incorporate the model into the proton treatment planning system, and validate the model/planning system using clinical data (unpublished data in proton therapy have shown unusual normal tissue toxicities).

### 4.2 Proton range uncertainty and proton portal-image/CT

Range is an important parameter in proton radiotherapy because it determines the position of the Bragg peak. A wrong range calculation would place the Bragg peak in a wrong location and consequently may deliver a high dose to the wrong location. The proton treatment planning system uses the CT image model to calculate the range. However, CT images are derived from the interactions of X-rays with the body and are the volumetric mapping of electron density in the body, while the range is also contributed significantly by the nuclear stopping power. Therefore, the range calculated from the X-ray-based CT images has an uncertainty, which was reported to be ±2.4% of the calculated range, plus an additional 1.2 mm uncertainty.^51^ In addition, a variable RBE may also shift the range slightly and therefore is another source of uncertainty.^51^ The range uncertainty is thus estimated to be ∼ 6 mm if the tumor is situated 20 cm from the entrancing surface. An additional planning target volume (PTV) margin is usually added to the target in currently clinical practice. However, adding a PTV margin greatly increases the dose to the normal tissues. Several technologies have been under research to overcome the range problem. These include using dual energy CT,^52^ position verification by PET imaging,^53^ gamma camera,^54^ and proton portal imaging,^55^ and using proton CT (pCT) for planning.^55^

Proton portal imaging and pCT would be considered as the same technology because they use the same hardware system, with pCT for treatment planning and proton portal imaging for range verification. In addition, the system can also be used for imaging-guided positioning. For simplicity, we refer this technology as pCT technique hereinafter. The pCT technology would be considered as the best choice and the focus of discussion here because it may fundamentally solve the range uncertainty problem. Two different approaches have been proposed for the pCT technology.^55^ The first is called proton integrating system. It basically uses a 2D imaging panel to measure the integrated dose in each pixel, which is similar to the X-ray based imaging panel. However, this approach appeared to have very poor spatial resolution due to multiple coulomb scatters of protons along their pathway. The second is called proton tracking system, which uses position sensitive detectors placed before and after the imaging object to detect the position of each proton, and hence estimate the most-likely path of each proton within the imaging object, and use a range telescope to measure the energy deposition for each proton. The tracking system greatly improved the spatial resolution in comparison to the integrating system. However, the use of multiple detectors before and after the imaging object greatly complicated the hardware setup, which may discourage its clinical application. In addition, the position detectors only detect the proton positions outside the imaging object. They may only provide limited information for the estimation of the most likely path of each proton inside the object, where the multiple coulomb scatters take place.

The author proposes here a third approach for the pCT technique. It uses a 2D proton energy detector panel, which can measure the residual energy of each proton exiting the imaging object at each pixel, and consequently the energy spectrum for each pixel. A narrow window in the energy spectrum of each pixel is determined as the first order estimation to calculate the water-equivalent path length (WEPL), and thus a first-order WEPL map is obtained for each projection. The first-order pCT is reconstructed from the WEPL maps of all projections. An iteration algorithm is then used to improve the pCT by including the multi-scatter process and information in the energy spectrum outside the narrow window. This technique may work better for pencil beam scanning machines than passive-scattering machines because in pencil-beam scanning, the entrance position of each proton is known so that the multi-scatter process in the imaging object can be better modeled. This technique not only have a simpler hardware setting for clinical application than the second approach, but also would have better image quality than the other two, because the narrow energy window can select those protons with minimal scattering angles and exclude protons with large multi-scatter angles.

## 5 SFRT

SFRT is a RT technique that have radiation fields (or a treatment volume) of highly-non-uniform and spatially oscillating dose distribution. SFRT was initially referred to as “grid therapy” because it initially utilized a grid to block discrete parts of radiation and achieve the highly-non-uniform and oscillating dose distribution. Grid therapy had been used clinically to treat deep-seated tumors in the era of orthovoltage and showed reasonably good treatment outcome.^56^ Grid therapy has also been used in LINAC-based MV X-rays to treat large tumors and showed encouraging results.^57^ However, the underlying mechanism for these clinical phenomena has been a puzzle for a long time, because the concept of SFRT is completely against the basic principle of conventional RT. In SFRT, a large amount of tumor volumes is not irradiated or severely underdosed, while in conventional RT, tremendous medical physics quality assurance efforts have been devoted to ensure sufficient dose coverage of the entire target volume, including the use of clinical target volume (CTV) and PTV. The establishment of immune system's role in RT may partially explain the reported success of the clinical application of SFRT, and support the use of SFRT technique over the conventional RT in the era of immunotherapy, especially for treating large tumors. In this section, we discuss the technological aspects of various SFRT approaches.

### 5.1 Single-field 1D/2D SFRT technique

Many SFRT approaches have been proposed. These approaches can be categorized according to the number of dimensions of dose oscillation in the target volume or the number of fields used to achieve the dose oscillation. Most of the SFRT techniques use a single field with a grid to achieve the goal. When the grid has only a one-dimensional oscillation pattern, such as a multi-slit collimator, the oscillation of dose distribution in the target is only in one dimension, and the SFRT technique can be considered as 1D SFRT technique. When the grid has a two-dimensional oscillation pattern, the dose distribution in the target volume would show oscillation in 2 dimensions, and the technique can be considered as a 2D SFRT technique. In addition, the multi-leave collimator (MLC) in a typical clinical LINAC can also be used to produce 2D oscillating dose distribution for SFRT. Due to its simplicity, the LINAC-based 2D SFRT technique is still frequently used in some clinics to treat large tumors.^58^ On the other hand, the 1D SFRT technique has been used in microbeams from high intensity synchrotron radiation source with relatively low X-ray energies (30-200 keV).^59^, ^60^ The microbeam SFRT has shown remarkable normal tissue sparing effect with reasonable tumor killing in preclinical studies.^59^, ^60^ However, the energy of the microbeam is relatively low so that its penetration is not favorable for clinical use. Consequently, minibeam with beam width in sub millimeter has been developed.^61^

### 5.2 Multi-field 3D SFRT technique

The single-field based 1D/2D SFRT technique sacrifices the advantage of dose conformation to the target in multi-field radiation delivery, so that the net gain by the single-field SFRT is limited. Multi-field based 3D SFRT technique can overcome this problem and still maintain the characteristics of the SFRT technique. A 3D SFRT approach was first proposed by *Wu et al.*, and was achieved by delivering focused radiation to several vertices inside the target using stereotactic radiotherapy techniques such as Cyberknife, Gammaknife or any other stereotactic techniques.^62^ The technique was also named as “lattice radiation” because these vertices form a lattice structure inside the target. However, when the number of vertices increases (for example to 20), the multiple small beams in different directions to different vertices intercept in locations other than the vertices, which may greatly degrade the oscillating dose distribution. In addition, the radiation delivery time and the complexity of the treatment plan increase with the number of vertices.

**Fig. 3:**
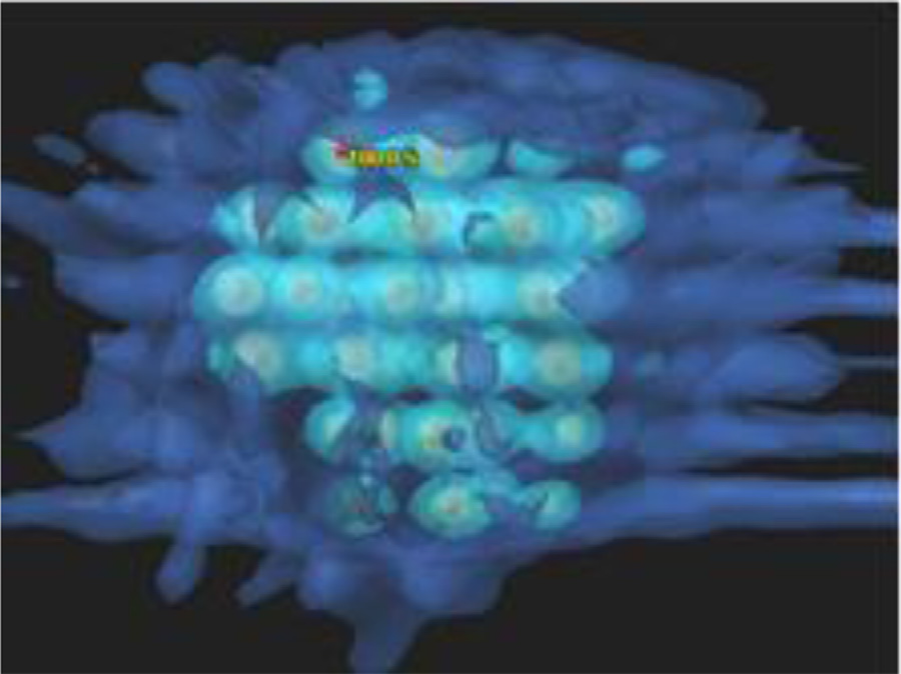
Three-dimensional (3D) dose distribution in a target and surrounding normal tissue using a 3D spatially fractionated radiotherapy technique by Jin et al.^55^ Very high dose in the spheres within the target, relatively low or intermediate dose in the rest of target volume, and low and oscillating doses in the surrounding normal tissues.

An MLC-based inversely optimized 3D SFRT technique was proposed to overcome this problem.^63^ The technique first defined multiple small spheres (typically more than 100) orderly distributed inside the gross tumor volume (GTV) to form a lattice. Radiation fields in channeling directions, in which the spheres were all perfectly aligned along a grid in their beam's eye views (BEV), were then chosen. The MLC-based intensity modulated inverse planning was finally used to maximize the radiation dose to the sphere, and minimize the dose to the rest of the volume in the GTV. As shown in Fig. 3, this 3D SFRT technique can deliver very high dose to the spheres inside the GTV, intermediate dose to the rest volume of GTV, and low and oscillating dose to normal tissues outside the GTV. To further reduce the radiation delivery time, grids corresponding to the pattern of spheres in each BEV can be used to replace the MLC segmentation.

### 5.3 Quasi 3D SFRT technique

Other SFRT approaches, which may not be categorized as single-field 1D/2D or multi-field 3D SFRT techniques, may be categorized as quasi-3D SFRT technique. One approach is to use multiple SFRT fields to achieve relatively uniform dose distribution in the target and oscillating dose distribution in the normal tissue outside the target. For example, two complimentary (interlaced) 1D-SFRT fields cross-fire to the target in different directions can achieve relatively uniform dose in the target.^64^ In addition, because some radiation beams (such as the very high-energy electrons) have relatively large penumbra than photons, and the penumbra increases with increasing depth, the oscillating dose pattern for a SFRT field is gradually flattened in the target region due to the large depth if the gaps between two opening beams are carefully selected.^65^ Multiple such SFRT fields in different directions can result in quite uniform dose in the target and highly oscillating dose distribution outside the target. However, this type of SFRT techniques lost the SFRT characteristics in the target, and may be similar to the classic IMRT technique in terms of relatively uniform dose in the target and modulating dose distribution outside the target. It is not clear whether the radiobiological effect to the tumor is favorable for the uniform dose distribution or for the oscillating dose distribution.

Another quasi-3D SFRT approach is to use a single proton 2D SFRT field to achieve 3D oscillating dose pattern in the target. This can be achieved by placing the Bragg peak at discrete depths with different proton energies. Multiple such proton 2D-SFRT fields from different directions can also be used to reduce the dose to surrounding normal tissues and improve the dose conformity to the target. Considering that FLASH RT, proton technique and 3D SFRT could all be used by this approach, this technology may need to be specifically singled out for future research and development in the era of immunotherapy.

## 6 Discussion and final remarks

We have presented FLASH RT, proton RT and SFRT as three special RT technologies that would have important application in the era of immunotherapy, because of their potential to minimize radiation dose and damage effect to the immune system, while maintaining a sufficient dose or tumor killing effect in the target. Certainly, their application should be coupled with fundamental researches on the radiobiology, cancer biology and immunology to better understand the underlying mechanism and detailed role of immune system in killing cancer cells during RT. For example, researches are required to study how radiation affects or damages the immune system, specifically, which immune cell subsets are responsible for the radiation-activated anti-tumor immunity, and the radiation effect on these subsets. It should be noted that besides these three technologies, other RT techniques are also able to achieve similar goals. These techniques include brachytherapy, intra-operative RT, and single-fraction or hypo-fractionated stereotactic radiosurgery or stereotactic body radiation therapy (SBRT) techniques. In addition, margin reduction techniques, such as simply eliminating PTV margins, using adaptive radiotherapy and image guidance to reduce PTV margin, may also significantly reduce the dose to the immune system.

Through its 125 years' history, RT has been demonstrated to be an effective cancer treatment modality. The reason that the RT technology is able to completely eliminate cancer cells from a body for various types of cancers and for many individuals is not only because the high dose radiation can kill sufficient cancer cells, but also because the body has a functional immune system to be re-activated to eliminate the remaining cancer cells. Future RT technologies are able to deliver higher radiation dose to the tumor while reducing the dose to the immune system. Therefore, it is expected that they can significantly improve the cancer treatment outcome in comparison to current RT technologies.

## Declaration of competing interest

The author declares that he has no conflict of interests.
